# Evaluation of the Impact of Cochlear Implantation on Patients’ Working Life: A Cross-Sectional Study

**DOI:** 10.3390/healthcare12050566

**Published:** 2024-02-29

**Authors:** Yüksel Olgun, Mehmet Emin Arayici, Armağan İncesulu, Ülkü Tuncer, Enis Alpin Güneri, Hülya Ellidokuz, Levent Olgun

**Affiliations:** 1 Department of Otorhinolaryngology, Faculty of Medicine, Dokuz Eylül University, 35220 İzmir, Türkiye; alpin.guneri@deu.edu.tr; 2 Department of Public Health, Faculty of Medicine, Dokuz Eylül Universtiy, 35220 İzmir, Türkiye; mehmet.e.arayici@gmail.com; 3 Department of Otorhinolaryngology, Faculty of Medicine, Osmangazi University, 26040 Eskişehir, Türkiye; armaganincesulu@yahoo.com; 4 Department of Otorhinolaryngology, Faculty of Medicine, Çukurova University, 01330 Adana, Türkiye; ulku.tuncer@acibadem.com; 5 Department of Biostatistics and Medical Informatics, Faculty of Medicine, Dokuz Eylül Universtiy, 35220 İzmir, Türkiye; hulyazeyda@gmail.com; 6 Department of Otorhinolaryngology, Faculty of Medicine, Başkent University, 06790 Ankara, Türkiye; levolgun52@gmail.com

**Keywords:** cochlear implants, work life, profound hearing loss

## Abstract

Hearing loss that arises from various causes at different stages of life has a direct impact on individuals’ physical and mental well-being. This paper aimed to evaluate the employment, workplace adaptation, productivity, and professional success of individuals who have hearing loss and whose hearing loss is corrected with a cochlear implant. In this cross-sectional study, data were collected between November 2022 and March 2023 with the participation of individuals with cochlear implants living in several settlements in all regions of Türkiye. A total of 142 participants with severe hearing loss who were corrected with a cochlear implant were included in this study. The survey method was used to collect data for the study. The questionnaire consisted of 32 questions and was distributed to the participants online. In the first part of the questionnaire (questions 1–10), the general characteristics of implant patients were investigated. In the second part (questions 11–32), the positive or negative effects of implantation on the work lives of the participants were evaluated. Almost half of the research group (49.3%, *n* = 70) consisted of women, and the mean age of the participants was determined to be 35.8 ± 14.8 years. There was no significant difference between gender, educational status, implanted side, working time, working style (physical, desk), and factors affecting work life (*p* > 0.05). Professional satisfaction and success at work increased significantly more in those with acquired hearing loss (*p* = 0.010). Post-implantation workplace compliance, success, and productivity were found to be higher in those with acquired hearing loss (*p* = 0.013). Hearing loss had a significantly less negative impact on work performance in those implanted in childhood than in those implanted in adulthood (*p* = 0.043). It was observed that hearing loss had a greater negative impact on the work life of married people (*p* = 0.006). Cochlear implantation greatly enhances workplace satisfaction, increases self-confidence, and has a positive impact on the future of profoundly deaf individuals.

## 1. Introduction

As is well documented by the World Health Organization (WHO), over 5% of the world’s population, which amounts to more than 430 million people, suffers from hearing loss (WHO, 2023 [1]). Hearing loss that arises from various causes at different stages of life has a direct impact on individuals’ physical and mental well-being [2,3,4,5]. A growing evidence base suggests that untreated congenital severe or profound hearing loss may also restrict individuals’ ability to hear, comprehend spoken language, acquire their native language, and develop speech skills using that language [2,3]. Moreover, these individuals not only face challenges in limited participation in the workforce but also encounter issues that lead to the termination of their professional lives due to barriers, resulting in significant workforce loss [6].

The cochlear implant (CI), which emerged in the late 20th century, has revolutionized the restoration of auditory function for individuals with severe to profound hearing loss [7,8,9]. A CI can facilitate the partial or significant recovery of individuals’ hearing abilities and enhance their communication with the surrounding environment. Individuals with congenital hearing loss who have received a CI are expected to receive education in related educational institutions and participate in society and working life. In the case of individuals who have developed severe to profound hearing loss later in life, cochlear implantation is anticipated to enable the acquisition of normal or near-normal hearing abilities, allowing them to reintegrate into social and professional working environments [10,11,12,13].

However, despite the rehabilitation of their hearing, speech, and language skills, individuals with varying degrees of impairment remain disabled to some extent. This can result in challenges when it comes to participating in or maintaining employment and may also affect work productivity [3,10,11,12,13]. There is limited research conducted worldwide on the participation of individuals with hearing disabilities and/or CI in the workforce, and there is a lack of studies conducted in our country as well [6,11,12,13,14]. Evaluating the challenges faced by individuals with implants in the workplace and assessing the data collected from these individuals is crucial for engaging with relevant non-governmental organizations, social security institutions, and the Ministry of Labor and Social Security to find solutions to these issues. Therefore, investigating the impact of this treatment on society will not only provide insights but also contribute to cost–benefit analyses.

The purpose of this paper was to investigate the employment rates, workplace adaptation, productivity, access to in-service training, and vocational achievements of individuals with severe to profound hearing loss who have undergone cochlear implantation to restore their hearing abilities.

## 2. Materials and Methods

The data for this study, which was planned and carried out as a cross-sectional type, were collected between November 2022 and February 2023. The research was planned at a center in İzmir, and individuals with cochlear implants in all settlements in Türkiye were expected to participate online. Before the collection of research data, ethical approval was obtained from the Dokuz Eylül University Non-Interventional Research Ethics Committee (GOEAK-7436). Similarly, permission was also obtained from the Association of Children with Bionic Ears and the Association of the Hearing Impaired and Their Families. Messages were sent to the social media accounts of the members of the association. In addition, social media platforms were used to reach individuals with cochlear implants.

Male and female patients who underwent unilateral or bilateral cochlear implantation in childhood and/or adulthood were included in the study. People who had brain stem implants and had additional disabilities other than hearing loss were excluded from the study. This includes significant visual impairments, major cognitive disorders, and severe motor impairments that would necessitate accommodations beyond those related to hearing loss.

The estimation for the sample size was carried out in the open-access OpenEpi program. It is estimated that there are approximately 2500 people with cochlear implants working actively in various corporate lines in Turkey. It was planned to reach a total of 155 participants at a 95% confidence level for 80% power and a 5% worst-case error margin. A total of 204 people responded to the surveys. Of these individuals, 62 participants who did not meet the inclusion criteria and had missing survey data (did not respond to more than 50% of the survey) were excluded from the study. A total of 142 participants with severe to profound hearing loss who were corrected with a cochlear implant were included in this research.

The survey consisted of a total of 32 questions and two parts. In the first part of the survey, which consisted of 10 questions evaluating information such as the cause, characteristics, and duration of hearing loss, the date of cochlear implantation, the time elapsed since the operation, and the patient’s educational status, the general characteristics of adult implanted patients were investigated. In the second part of the questionnaire (questions 11–32), there were questions aiming to reveal the positive or negative effects of implantation on participants’ work lives. The questionnaire was delivered to the participants online, and informed consent was obtained from the individuals before the survey was conducted. After the data were collected online, they were transferred to Microsoft Excel and then to the SPSS program for data analysis.

The independent variables of the study were determined as follows: age, sex, education status, the time elapsed after implantation, marital status, cause of hearing loss, type of hearing loss (congenital hearing loss and acquired hearing loss), first implant placement (implanted in childhood (<12 years old) vs. implanted in adulthood (≥12 years old)), implanted ear, number of implants used, whether or not the participant worked at a job, duration of work at a job, and the participant’s work line. The dependent variables of the study were as follows: the negative effect of hearing loss on job performance, time spent with verbal communication after implantation, how implantation affects communication in the workplace, whether there is discrimination at work due to the implant, how implantation affects performance at work, how to worry about job loss changes after implantation, success at work and job satisfaction after implantation, how income has changed after surgery, how self-confidence has been affected after implantation, how implantation has impacted work fit and productivity, and how implantation has impacted prospects for work.

### Statistical Analysis

The conformity of continuous variables to normal distribution was evaluated by Kolmogorov Smirnov and Shapiro Wilk tests and skewness and kurtosis symmetric distributions. Continuous variables suitable for normal distribution were reported as mean and standard deviation (SD), and variables not suitable for normal distribution were reported as median and interquartile range (IQR). The chi-square test was used in the analysis of categorical variables, and the Fisher exact test was utilized when necessary. Statistical significance was quantified at the two-tailed *p* < 0.05 level. All statistical analyses were performed using the SPSS (v29) package program.

## 3. Results

A total of 142 patients with hearing loss (acquired/congenital hearing loss) who received one or two cochlear implants were included in this study. Almost half of the research group (49.3%, *n* = 70) consisted of women (Table 1). The mean age of the participants in the study was determined to be 35.8 ± 14.8 years. Hearing loss was acquired (childhood, progressive, and adulthood) in 56.3% (*n* = 80) of the individuals. The implant was unilateral in 87.3% (*n* = 124) of the study group, and there were 18 (12.7%) bilateral cases. Other descriptive features of the study group are available in Table 1.

The effect of the cochlear implant on work life was evaluated through the answers given to the questionnaire. Accordingly, there was no significant difference between the genders in terms of the negative effect of hearing loss on job performance, time spent with verbal communication after implantation, how implantation affects communication in the workplace, whether there is discrimination at work due to the implant, how implantation affects performance at work, how worry about job loss changes after implantation, success at work and job satisfaction after implantation, how income has changed after surgery, how self-confidence has been affected after implantation, how implantation has impacted work fit and productivity, and how implantation has impacted future prospects for work (*p* > 0.05). Similarly, no significant difference was found between the related variables and the implanted side, educational status, working time, working style (physical, desk), and whether or not he/she had a regular job (*p* > 0.05).

The effect of the type of hearing loss (congenital vs. acquired hearing loss) on the working life of the participants was examined, as presented in Table 2. It was concluded that those with acquired hearing loss had significantly more negative work performance than those with congenital hearing loss (*p* = 0.027). The time elapsed with verbal communication post-implantation and general communication at work increased more in those with acquired hearing loss, and the difference between them was statistically significant (*p* = 0.015, *p* = 0.006, respectively). Similarly, professional satisfaction and success at work increased significantly more in those with acquired hearing loss (*p* = 0.010) (Table 2). In addition, it was determined that post-implantation workplace compliance, success, and productivity increased more in those with acquired hearing loss (*p* = 0.013). There was no significant difference between the other variables and the type of hearing loss.

The effect of the time of first implant placement (implanted in childhood vs. implanted in adulthood) on the participants’ working lives was scrutinized, as shown in Table 3. It was concluded that individuals implanted during childhood experienced a less negative effect of hearing loss on their work performance compared to those who received implants in adulthood, with a statistically significant difference (*p* = 0.043). Inversely, the time elapsed with verbal communication post-implantation increased more in those implanted in adulthood than in those implanted in childhood, and the difference between them was statistically significant (*p* = 0.040). There was no notable difference between the other variables and the time of the first implant placement (*p* > 0.05).

The effect of the implant side (bilateral vs. unilateral cases) on the working lives of the participants was also investigated. There was no significant difference between the variables and the implant side.

The relationship between the duration of working with hearing loss before implantation and success at work and professional satisfaction after implantation was evaluated. As seen in Table 4, it has been observed that professional satisfaction and success at work increase more in those who have been working with hearing loss for 5 years or more before implantation (*p* = 0.037). 

When marital status and the negative effect of hearing loss on work performance were also considered, it was observed that hearing loss had a greater negative impact on the work lives of married people (*p* = 0.006) (Table 4). There was no significant difference between other variables and marital status (*p* > 0.05). 

## 4. Discussion

It is well documented that there are numerous studies on the remarkable benefits of cochlear implant treatment in improving the quality of life for individuals with hearing impairments [15,16,17,18]. In contrast, there are relatively few reports on the benefits of cochlear implants and their impact specifically in terms of individuals who are actively employed or engaged in the workforce. In this current research, a total of 142 participants with hearing loss (acquired/congenital) who had received one or two CIs were investigated in terms of employment status, workplace adaptation, productivity, and vocational achievements.

In a study conducted by Kós et al., the impact of cochlear implants on the professional and vocational lives of hearing-impaired adults was investigated [11]. A total of 67 patients were included in the study. Out of the 67 patients examined, 50.7% (*n* = 34) were actively employed at the time of implantation. Following the implantation, 29 of these patients remained active in the workforce. After implantation, four individuals reported positive developments in their careers. In our study, out of a total of 81 patients who reported being involved in regular or irregular employment, 6 individuals lost their jobs after implantation. Additionally, one patient stated that they had transitioned to a worse position in their former workplace. Of these individuals, 14.8% reported that their position in their former workplace changed positively after implantation. Therefore, these results suggest that cochlear implantation has a positive impact on changes in workplace positions. 

A study conducted by Huarte included a total of 60 individuals, with 50 of them being actively employed, focusing on hearing loss corrected with cochlear implants [6]. The researchers reported that 94.2% of the included participants were satisfied with their current jobs, and almost all of them (93.0%) stated that they were more motivated to go to work after implantation. Additionally, in the study, it was reported that the majority of individuals (79.3%) perceived themselves as more competent after the surgery and device activation [6]. In this study, 73.6% of the individuals reported an improvement in their job performance after implantation. Furthermore, it was found that approximately three-quarters of the individuals experienced an increase in job success and professional satisfaction after implantation. Taken together, these results allow the statement that individuals who underwent implantation showed a notable increase in workplace performance, workplace motivation, and professional satisfaction.

Another critical issue to discuss is the impact that the timing of cochlear implantation has on the working lives of individuals. Cochlear implants received in childhood and adulthood may show differences in performance as expected. Findings from various studies have shown that early implantation has more positive effects on auditory performance [19,20]. In our study, those receiving their implants in childhood reported more positive work performance compared to their counterparts implanted in adulthood. This could be attributed to the critical processes of auditory and language development that are optimally exploited in childhood, facilitating smoother integration into communication-intensive work environments. Moreover, the enhanced adaptability to verbal communication in individuals implanted during childhood could contribute to a smoother transition into various occupational roles, underscoring the importance of early intervention for individuals with hearing impairments. These findings support policies and practices that support timely access to cochlear implantation for children to optimize their future work performance and quality of life. The differential impact of cochlear implantation timing on verbal communication skills advancement post-implantation, as highlighted by our findings, offers insights into the adaptative capacity of individuals with cochlear implants. The adaptability in adults, likely driven by their more mature cognitive and motivational resources, demonstrates that while early implantation is advantageous for mitigating the negative impacts of hearing loss on work performance, adults also stand to gain substantially in terms of verbal communication skills following implantation. However, this increased time elapsed with verbal communication improvement in adults implanted in adulthood also reflects the challenges they face in compensating for a lifetime of hearing loss in terms of verbal communication skills. Therefore, this suggests that early implanted individuals may integrate more seamlessly into work environments. It may also show that adults spend more time on verbal communication in professional environments to benefit from the full potential of their cochlear implants. 

In several studies, it has been proposed that patients with bilateral implants observed notable improvements in their perceived hearing capabilities and overall life quality, prominently exceeding the benefits experienced by users of unilateral implants [3,21,22,23]. Our study revealed that the implant side did not have a significant effect on various factors of working life. However, the number of participants with bilateral implants in our study was quite small. Therefore, the lack of a significant difference suggested that it might be related to the number of participants. It should be underlined that there is an increasing need for research on the subject with a larger number of participants, including individuals with unilateral and bilateral implants. 

In this current research, it was observed that individuals with acquired hearing loss experienced a significant increase in professional satisfaction and job success, and after implantation, workplace adaptation, success, and productivity showed a greater increase. These results suggest that individuals with acquired hearing loss adapt more quickly to the workplace compared to those with congenital hearing loss. Furthermore, while the study contributes valuable insights into the under-researched area of cochlear implant recipients in the workforce, several limitations must be acknowledged. One of the limitations of this study is that the data were collected through surveys. The participants were directed to complete the survey online and fill it out on their own. This raises the possibility of a bias in the responses given to the survey questions, either in a positive or negative direction. The study’s design and the specific population sampled limit the generalizability of the findings. Another limitation of the study is a self-selection bias. People who greatly benefit from cochlear implants might be more willing to self-select to participate in the study. Therefore, the potential impact of self-selection bias on our results highlights the need for cautious interpretation of the findings. According to the general information we received from the organizations, we determined that the demographic data of 2500 individuals with cochlear implants working in various business lines had similar demographic characteristics to the population in our study. Additionally, the prevalence of acquired hearing loss and congenital hearing loss was approximately similar in 2500 people. Thus, by minimizing this bias, we increased the reliability of the findings of our study. The lack of broader data that can be applied to a wider population of individuals with hearing disabilities or cochlear implants means that our results might not be representative of all such individuals in the workforce. Another limitation is that some participants left certain questions unanswered, leading to their exclusion from the study. Although the demographic data and hearing loss types of these people are similar to the population we included in the study, this may have hindered the attainment of stronger results. These limitations highlight the need for incorporating more robust and comprehensive methodologies in future research, including using standardized measures and larger, more diverse populations, to enhance the applicability and impact of the findings.

## 5. Conclusions

Herein, the employment status, workplace adaptation, productivity, and vocational achievements of individuals with acquired hearing loss and congenital hearing loss were investigated. Cochlear implantation significantly enhances workplace satisfaction, increases self-confidence, and has a positive impact on the future of individuals with severe hearing loss by promoting their communication abilities. It is highly recommended and of critical importance to design studies related to the topic on a larger scale, encompassing a broader range of participants. Moreover, considering the limited number of studies in the field, research covering different samples and groups on the topic will make significant contributions to the literature in this field.

## Figures and Tables

**Table 1:** Distribution of the participants according to demographic, academic, and descriptive features.

Variables	Total (*n* = 142)
Age (years), mean ± sd	35.89 ± 14.80
Sex, *n* (%)	
Women	70 (49.3)
Men	72 (50.7)
Education status, *n* (%)	
High school and below	63 (44.4)
University and above	79 (55.6)
Marital status, *n* (%)	
Married	66 (46.5)
Single or divorced	76 (53.5)
Type of hearing loss, *n* (%)	
Congenital	62 (43.7)
Acquired hearing loss in childhood	46 (32.4)
Progressive	4 (2.8)
Acquired hearing loss in adulthood	30 (21.1)
Implant side, *n* (%)	
Unilateral	124 (87.3)
Bilateral	18 (12.7)
Implant usage time, *n* (%)	
<5 years	42 (30)
≥5 years	98 (70)
First cochlear implant age, *n* (%)	
<4 year	17 (12.0)
4–12 years	28 (19.9)
>12	96 (68.1)
Working time, *n* (%)	
0–10 years	51 (49.0)
>10 years	53 (51.0)
Working time with hearing loss before implant, *n* (%)	
<5 years	54 (51.4)
≥5 years	51 (48.6)
Workplace, *n* (%)	
Public sector	48 (46.6)
Private sector	50 (48.5)
Own working place	5 (4.9)
Working status at the date of implantation	
No regular job	46 (44.2)
Have a regular job	58 (55.8)
Working status after the implantation	
I continued my previous job	42 (51.9)
My position has changed positively	12 (14.8)
My position has changed negatively	1 (1.2)
I changed my job	20 (24.7)
I lost my job	6 (7.4)
Type of work, *n* (%)	
Physical work	9 (6.3)
Desk work	50 (35.2)
Other	83 (58.5)
Income after implantation, *n* (%)	
Decreased	9 (8.2)
Unchanged	66 (60.0)
Increased	35 (31.8)
How did implantation affect performance at work?	
Decreased or unchanged	29 (26.4)
Increased	81 (73.6)
How has success at work and professional satisfaction changed after implantation?	
Decreased or unchanged	23 (22.3)
Increased	80 (77.7)

**Table 2:** The effect of the type of hearing loss on the working lives of the participants.

Groups	Variables		*p* Value
Negative effect of hearing loss on work performance			
Type of hearing loss	Most of the time, *n* (%)	Sometimes or never, *n* (%)	
Congenital	16 (30.2)	37 (69.8)	0.027 ^a^*
Acquired hearing loss	35 (50.0)	35 (50.0)	
Time spent with verbal communication after implantation			
	Decreased or unchanged, *n* (%)	Increased, *n* (%)	
Congenital	13 (33.3)	26 (66.7)	0.015 ^a^*
Acquired hearing loss	9 (13.4)	58 (86.6)	
How implantation impacted communication in the workplace			
	Decreased or unchanged, *n* (%)	Increased, *n* (%)	
Congenital	11 (24.4)	34 (75.6)	0.006 ^a^*
Acquired hearing loss	4 (6.2)	61 (93.8)	
Do you experience discrimination at work because of your implant?			
	Often or always, *n* (%)	Rarely or never, *n* (%)	
Congenital	4 (7.8)	47 (92.2)	0.433 ^a^
Acquired hearing loss	8 (12.3)	57 (87.79)	
How has the worry of job loss changed after implantation?			
	Decreased or unchanged, *n* (%)	Increased, *n* (%)	
Congenital	34 (82.9)	7 (17.1)	0.383 ^a^
Acquired hearing loss	49 (89.1)	6 (10.9)	
How has success at work and professional satisfaction changed after implantation?			
	Decreased or unchanged, *n* (%)	Increased, *n* (%)	
Congenital	15 (34.9)	28 (65.1)	0.010 ^a^*
Acquired hearing loss	8 (13.3)	52 (86.7)	
How has income changed after surgery?			
	Decreased or unchanged, *n* (%)	Increased, *n* (%)	
Congenital	31 (70.5)	13 (29.5)	0.676 ^a^
Acquired hearing loss	44 (66.7)	22 (33.3)	
How was self-confidence affected after implantation?			
	Decreased or unchanged, *n* (%)	Increased, *n* (%)	
Congenital	12 (23.5)	39 (76.5)	0.077 ^a^
Acquired hearing loss	9 (11.7)	68 (88.3)	
How has implantation affected work compliance and productivity?			
	Decreased or unchanged, *n* (%)	Increased, *n* (%)	
Congenital	13 (26.0)	37 (74.0)	0.013 ^a^*
Acquired hearing loss	6 (9.0)	61 (91.0)	

^a^ Chi-square test, * statistically significant.

**Table 3:** The effect of the time of first implant placement on the working lives of the participants.

Groups	Variables		*p* Value
Negative effect of hearing loss on work performance			
First implant placement	Most of the time, *n* (%)	Sometimes or never, *n* (%)	
Implanted in childhood	9 (26.5)	25 (73.5)	0.043 ^a^*
Implanted in adulthood	41 (46.6)	47 (53.4)	
Time spent with verbal communication after implantation			
	Decreased or unchanged, *n* (%)	Increased, *n* (%)	
Implanted in childhood	8 (36.4)	14 (63.6)	0.040 ^b^*
Implanted in adulthood	13 (15.7)	70 (84.3)	
How implantation impacted communication in the workplace			
	Decreased or unchanged, *n* (%)	Increased, *n* (%)	
Implanted in childhood	6 (24.0)	19 (76.0)	0.085 ^b^
Implanted in adulthood	8 (9.5)	76 (90.5)	
Do you experience discrimination at work because of your implant?			
	Often or always, *n* (%)	Rarely or never, *n* (%)	
Implanted in childhood	2 (6.9)	27 (93.1)	0.728 ^b^
Implanted in adulthood	10 (11.6)	76 (88.4)	
How has the worry of job loss changed after implantation?			
	Decreased or unchanged, *n* (%)	Increased, *n* (%)	
Implanted in childhood	17 (85.0)	3 (15.0)	1.000 ^b^
Implanted in adulthood	65 (86.7)	10 (13.3)	
How has success at work and professional satisfaction changed after implantation?			
	Decreased or unchanged, *n* (%)	Increased, *n* (%)	
Implanted in childhood	6 (25.0)	18 (75.0)	0.742 ^a^
Implanted in adulthood	17 (21.8)	61 (78.2)	
How has income changed after surgery?			
	Decreased or unchanged, *n* (%)	Increased, *n* (%)	
Implanted in childhood	17 (70.8)	7 (29.2)	0.727 ^a^
Implanted in adulthood	57 (67.1)	28 (32.9)	
How was self-confidence affected after implantation?			
	Decreased or unchanged, *n* (%)	Increased, *n* (%)	
Implanted in childhood	7 (20.6)	27 (79.4)	0.457 ^a^
Implanted in adulthood	14 (15.1)	79 (84.9)	
How has implantation affected work compliance and productivity?			
	Decreased or unchanged, *n* (%)	Increased, *n* (%)	
Implanted in childhood	5 (16.1)	26 (83.9)	0.965 ^a^
Implanted in adulthood	14 (16.5)	71 (83.5)	

^a^ Chi-square test, ^b^ Fisher exact test, * statistically significant.

**Table 4:** The effect of marital status and duration of working with hearing loss before implantation on the working lives of the participants.

Groups	Variables		*p* Value
Negative effect of hearing loss on work performance			
Marital status	Most of the time, *n* (%)	Sometimes or never, *n* (%)	
Married	32 (54.2)	27 (45.8)	0.006 ^a^*
Single or divorced	19 (29.7)	45 (70.3)	
Time spent with verbal communication after implantation			
Marital status	Most of the time, *n* (%)	Sometimes or never, *n* (%)	
Married	9 (15.8)	48 (84.2)	0.174 ^a^
Single or divorced	13 (26.5)	36 (73.5)	
Do you experience discrimination at work because of your implant?			
Marital status	Most of the time, *n* (%)	Sometimes or never, *n* (%)	
Married	8 (14.5)	47 (85.5)	0.158 ^a^
Single or divorced	4 (6.6)	57 (93.4)	
Negative effect of hearing loss on work performance			
Duration of working with hearing loss before implantation	Most of the time, *n* (%)	Sometimes or never, *n* (%)	
0–5 years	21 (39.6)	32 (60.4)	0.456 ^a^
>5 years	23 (46.9)	26 (53.1)	
How has success at work and professional satisfaction changed after implantation?			
Duration of working with hearing loss before implantation	Decreased or unchanged, *n* (%)	Increased, *n* (%)	
0–5 years	14 (31.1)	31 (68.9)	0.037 ^a^*
>5 years	6 (13.0)	40 (87.0)	
How implantation impacted communication in the workplace			
Duration of working with hearing loss before implantation	Decreased or unchanged, *n* (%)	Increased, *n* (%)	
0–5 years	17 (35.4)	31 (64.6)	0.066 ^a^
>5 years	9 (18.8)	39 (81.3)	

^a^ Chi-square test, * statistically significant.

## Data Availability

The datasets used and/or analyzed in this study are available upon reasonable request from the corresponding author.
